# Supplementary material for: Dysfunction of the key ferroptosis-surveilling systems hypersensitizes mice to tubular necrosis during acute kidney injury
Source: Nat Commun. 2021 Jul 20;12:4402. doi: 10.1038/s41467-021-24712-6 (PMC8292346; doi:10.1038/s41467-021-24712-6)
Supplement: Supplementary file 1 — Supplementary Information [file 41467_2021_24712_MOESM1_ESM.pdf]

Supplementary Information to

Dysfunction of the key ferroptosis-surveilling systems hypersensitizes mice to massive tubular necrosis during acute kidney injury

Supplementary Methods

REAGENT or RESOURCE	SOURCE	IDENTIFIER	DILUTION
Antibodies			
Anti-4 Hydroxynonenal antibody	Abcam	Cat# ab46545	1:500
Rabbit monoclonal anti-RIPK1 (phospho S166), human	Cell Signaling	Cat# 65746	1:1000
Rabbit monoclonal anti-MLKL (phospho S358), human	Abcam	Cat# ab187091	1:1000
Rabbit polyclonal anti-MLKL, human	Genetex	Cat# GTX107538	1:1000
β-actin	Cell Signaling	Cat# 3700S	1:1000
Anti-mouse IgG; HRP-linked antibody	Cell Signaling	Cat# 7076S	1:5000
Anti-rabbit IgG; HRP-linked antibody	Cell Signaling	Cat# 7074S	1:5000

Supplementary table 1: Antibodies used in this study as well as the respective distributor and catalogue number.

REAGENT or RESOURCE	SOURCE	IDENTIFIER
Compounds & chemicals		
Erastin (type 1 FIN)	Sigma Aldrich	Cat# E7781
RSL3 (type 2 FIN)	Selleck Chemicals	Cat# S8155
FIN56 (type 3 FIN)	Sigma Aldrich	Cat# SML1740
FINO2 (type 4 FIN)	Keith Wörpel, Brent Stockwell	N/A
Ferroptocide (thioredoxin reductase inhibitor)	Paul Hergenrother	N/A
Birinapant (smac mimetic)	Chemietek	Cat# CT-BIRI
Ferrostatin-1 (Fer-1)	Merck Millipore	Cat# 341494
Necrostatin-1s (Nec-1s)	Merck Millipore	Cat# 5.04297.0001
human TNFα	BioLegend	Cat# 570108
zVAD-fmk	BD Biosciences	Cat# 550377
SYTOX Green	Life Technologies	Cat# S7020
7-AAD	BD Biosciences	Cat# 559925
Annexin-V-FITC	BD Biosciences	Cat# 556420
Annexin-V binding buffer	BD Biosciences	Cat# 556454
ITS Liquid Media Supplement (100x)	Sigma Aldrich	Cat#I3146
Kits		
LDH release assay	Promega	Cat# G1780
Bradford assay	Fisher Scientific	Cat# 23225

ECL™ Prime Western Blotting System	Fisher Scientific	Cat# GERPN2232
CellTiterGlo® Luminescent Cell Viability Assay	Promega	Cat# G7570
TUNEL	Roche	Cat# 11684795910

Supplementary Table 2: Compounds, chemicals and commercial kits used in this study.

Equipment		
Gradient HPLC system	Shimadzu	
Innova 4080 Incubator Shaker	New Brunswick Scientific, USA	
Fixed Speed Vortex Mixer “IKA Lab Dancer”	IKA®-Werke GmbH & Co. KG, Germany	IP-40
Dispersing system. The Bullet Blender®	Next Advance, USA	model BB24-AU
Ultrasonic bath	Daihan, Korea	WUC-A03H
Water purification system Millipore Milli-Q Gradient A10	Millipore, France	
Environmental Incubator Shaker G24; Digital Refrigerated Incubator/Shaker Innova 4330	New Brunswick Scientific, USA	
Nitrogen generator N2-04-L1466, nitrogen purity 99%+	Whatman	
MS/MS detector API 3000 with TurboIonSpray Electrospray module	PE Sciex, Canada	
Zeiss Axio Imager.A2,	Zeiss GmbH, Germany	
Promega GloMax®	Promega, Madison, USA	

Supplementary Table 3: Instruments and equipment used in this study.

Supplementary Figures and Legends

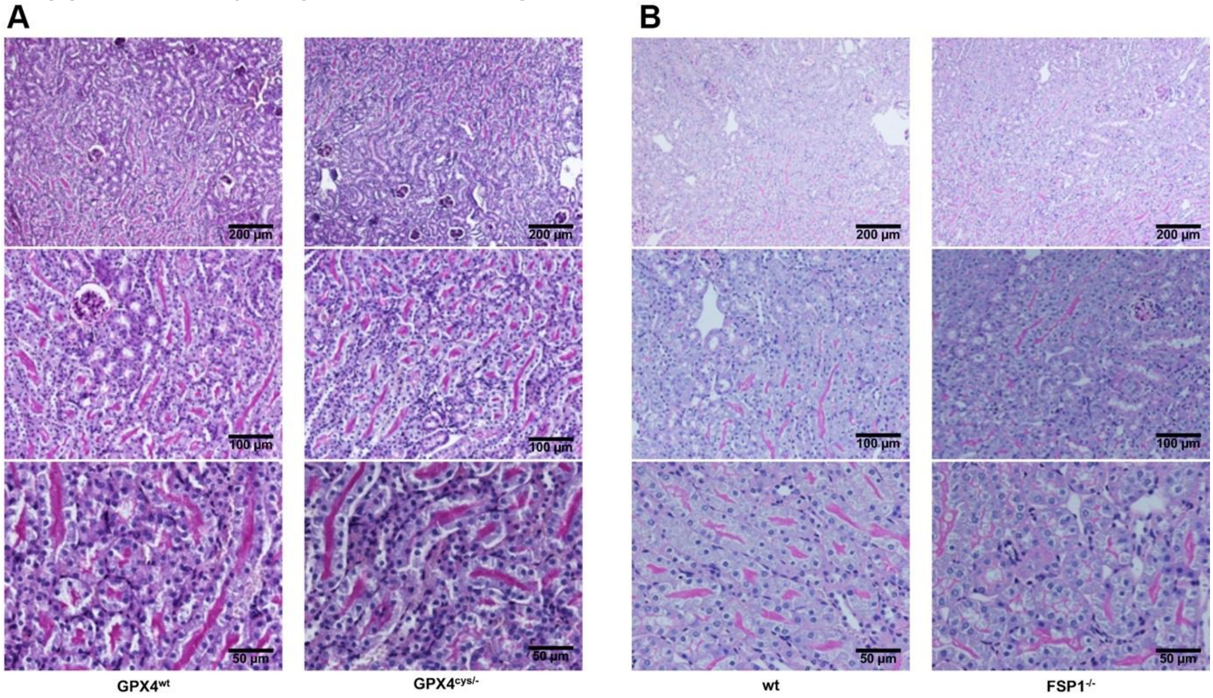

Figure S1: Untreated *Gpx4*$^{cys/-}$ and untreated *Fsp1*-deficient control kidneys. (A) *Gpx4*$^{cys/-}$ mice were induced to drive the Cre recombinase for 10 days. Note that the dysfunction of GPX4 in murine kidneys does not cause structural renal damage in different magnifications of PAS-stained renal sections in this setting. (B) Untreated 10-week-old *Fsp1*-deficient mice were stained for PAS. No structural damage is evident.

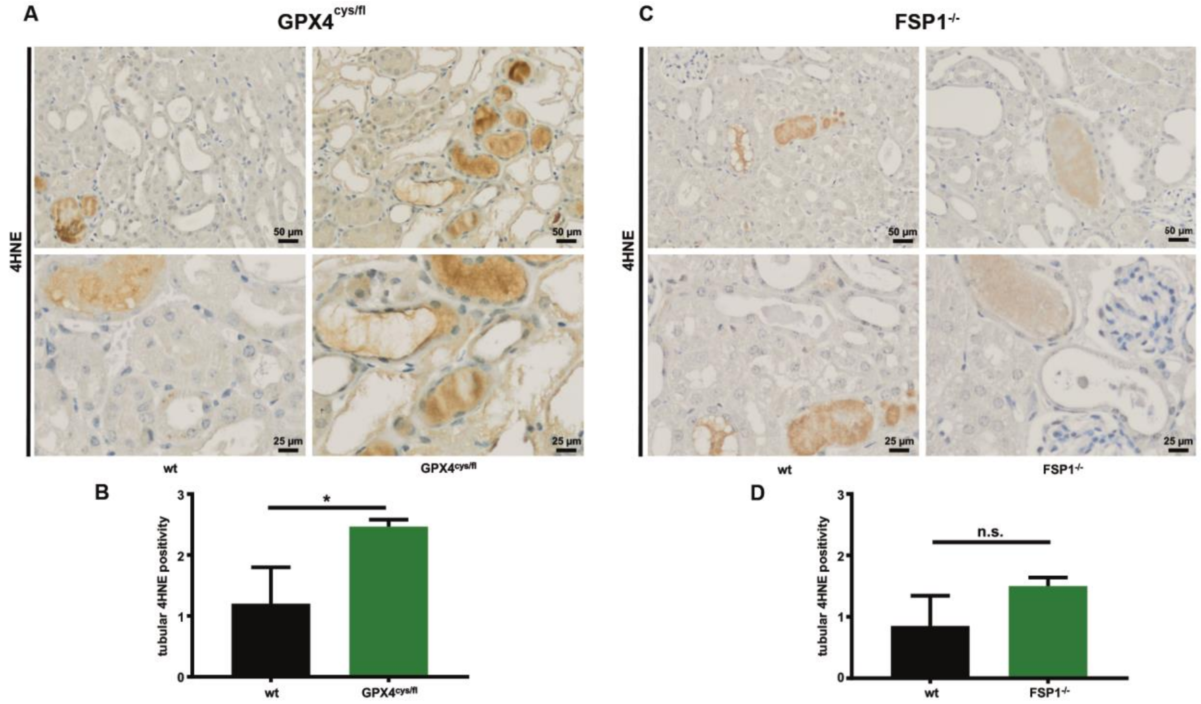

Figure S2 (corresponds to Figure 1): 4-Hydroxynonenal (4HNE) staining in Gpx4$^{cys/fl}$-and FSP1-deficient mice. Mice underwent renal ischemia-reperfusion injury as in Fig. 1. (A) 4HNE staining of kidney sections of wt littermates of Gpx4$^{cys/-}$-mice and (B) semiquantitative assessment of positivity. (C) Kidney sections of wild type littermates and FSP1-deficient mice were stained for 4HNE and (D) scored on a semiquantitative scale by an experienced nephropathologist. Bar graphs represent mean +/- SD. Statistics were calculated using student´s t-test.

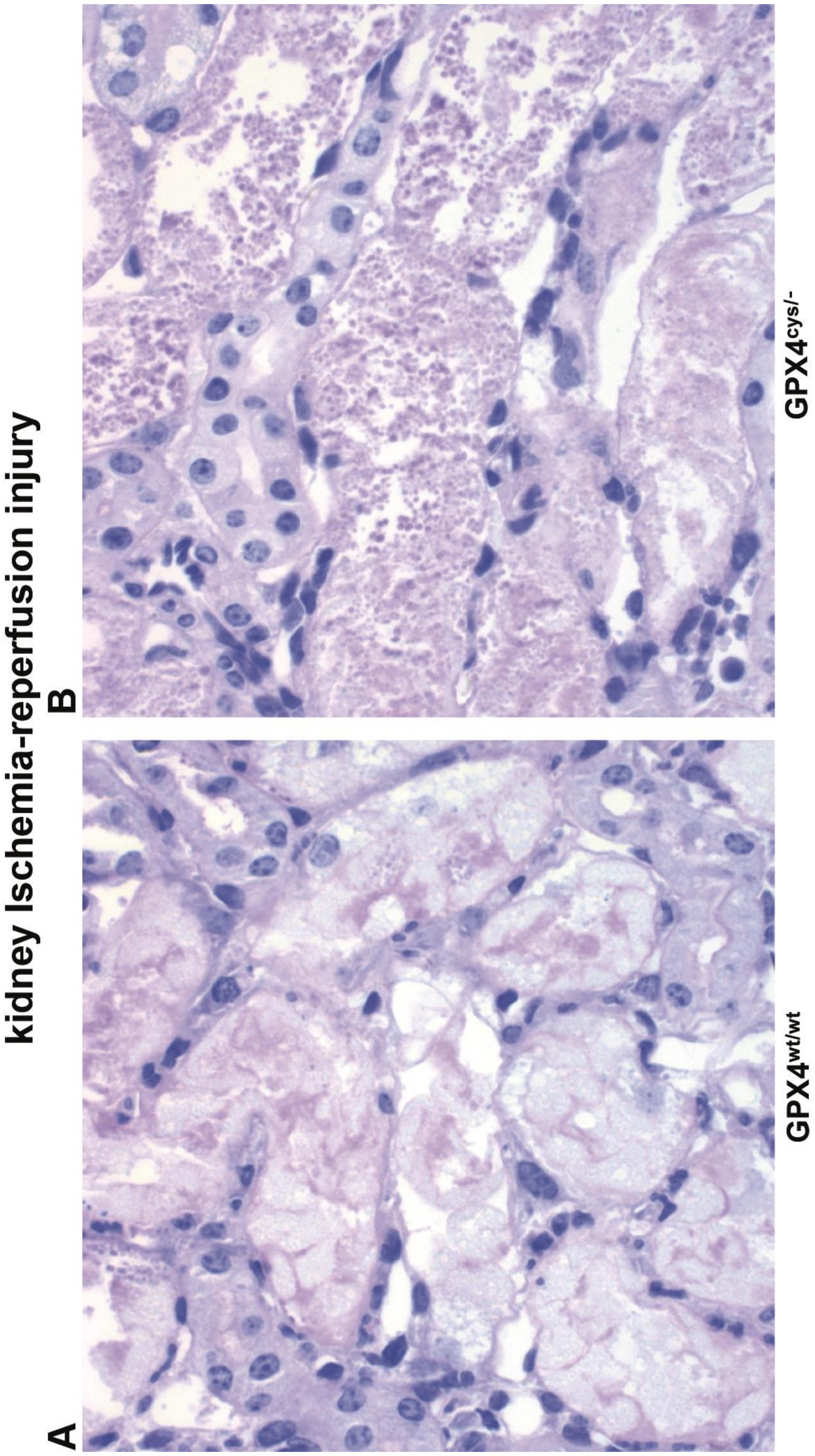

Figure S3: Acute tubular necrosis in *Gpx4*$^{cys/-}$ mice. Mice underwent standard ischemia-reperfusion injury as demonstrated in detail in Fig. 1. High-resolution images of *Gpx4*$^{wt/wt}$ (A) and *Gpx4*$^{cys/-}$ mice (B) are presented. Note the multiple vesicular fragmentation of entire tubules in the *Gpx4*$^{cys/-}$ mice.

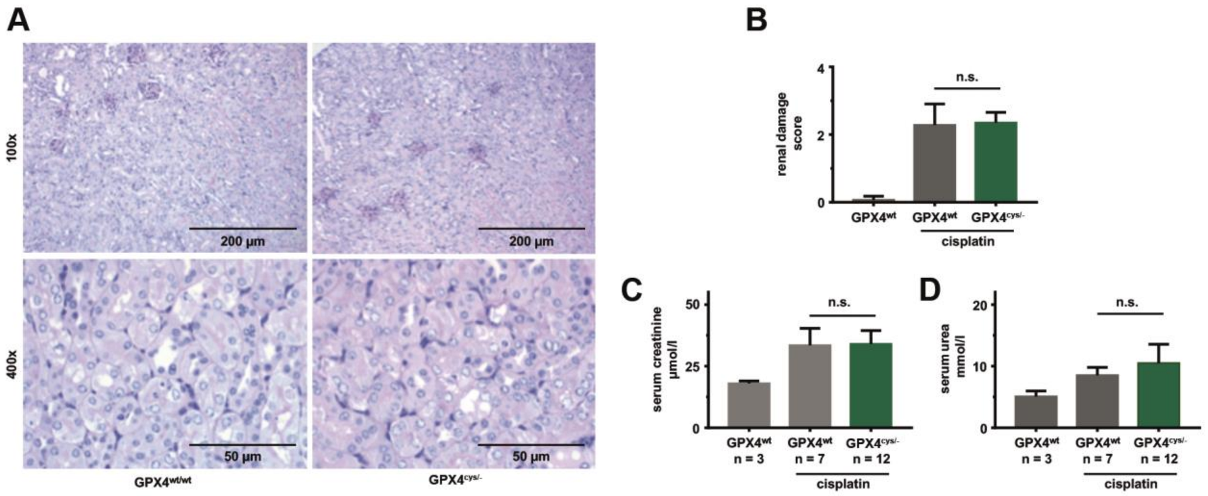

Figure S4: Renal cisplatin toxicity is unaffected by GPX4 dysfunction. *Gpx4*$^{cys/-}$ and *Gpx4*$^{wt/wt}$ mice were induced with tamoxifen as explained in detail in the methods section. A single dose of 20 mg/kg body weight cisplatin was injected intraperitoneally. (A) Typical toxic renal damage was visualized by periodic acid-Schiff (PAS)-staining and quantified using the renal damage score (B). Serum concentrations of creatinine (C) and urea (D) were measured 48 h following the injection of cisplatin. Note that no notably significant differences were detected in these assays between these genotypes. Bar graphs represent mean +/- SD. Statistics were calculated using student´s t-test.

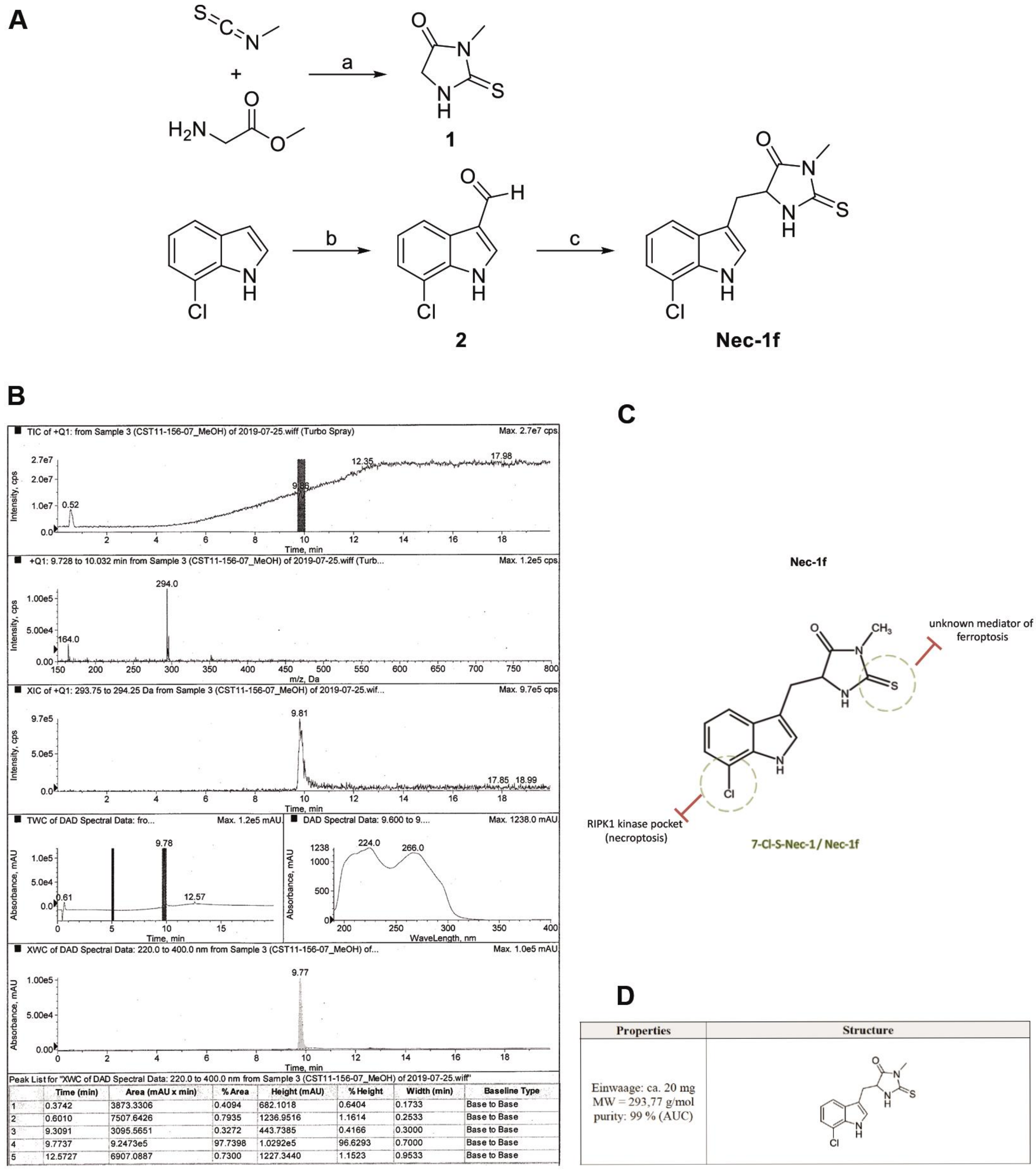

Figure S5: Synthesis of Nec-1f. (A) Synthesis of the dual-active compound Nec-1f. Reagents and conditions: (a) Et_3N, Et_2O, rt, 72 h; (b) $POCl_3$, DMF, rt, 3 h; (c) dithiothreitol, N-methylmorpholine, 110°C, 8 h. Nec-1f was synthesized using a newly developed convergent synthetic route. The thiohydantoin building block (1), which was obtained from methyl isothiocyanate and glycine methyl ester, was condensed with 7-chloro-1*H*-indole-3-carbaldehyde (2) in a Knoevenagel reaction. The presence of dithiothreitol did not only prevent the undesired dimerization of Nec-1f *via* disulfide bonds, but also provided a sufficiently high redox potential to reduce the initial double bond of the Knoevenagel product. (B) Comparison of the starting material and raw product by mass spectrometry. (C) Proposed mechanism of action of Nec-1f. Note that the chlorine residue on the indole ring structure is identical compared to Nec-1s, while replacement of oxygen with sulfur in the thiohydantoin moiety is the most likely structure responsible for the inhibition of ferroptosis. (D) MW, purity and structure of Nec-1f.

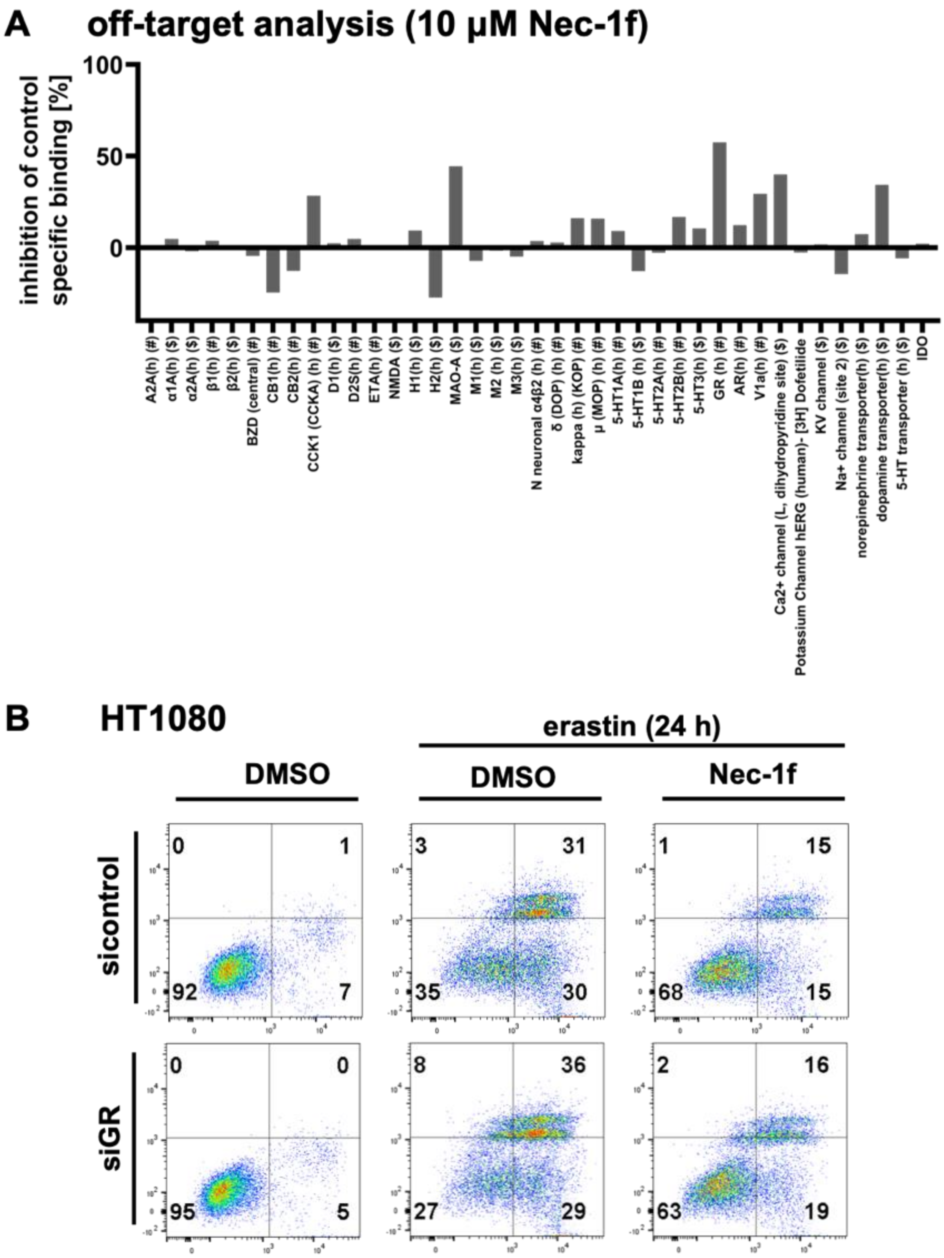

Figure S6: Off-target analysis of Nec-1f. (A) Nec-1f (10 µM) was tested in a commercially available safety screen analysis against common potential off-targets. Nec-1f binding was calculated as percent inhibition of the binding of a radioactively labeled ligand specific for each target. (B) Assessment of the efficacy of Nec-1f (30 µM) to protect HT1080 cells treated with siRNA against the glucocorticoid receptor (siGR) from erastin (5 µM)-induced cell death. Note that Nec-1f still prevents the cells from ferroptosis.

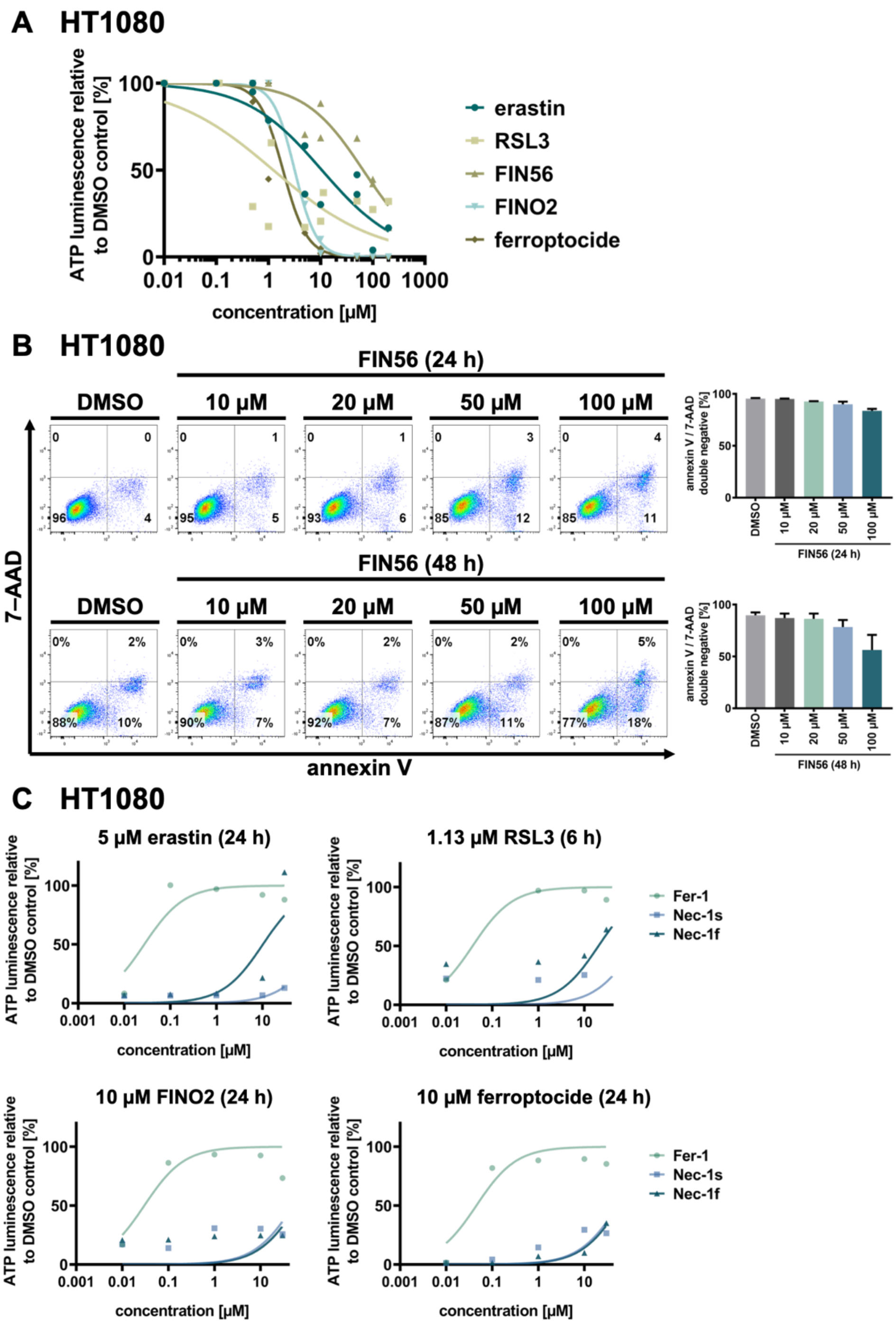

Figure S7: Dose response curves for all ferroptosis inducers (FINs) and ferroptocide and inhibitors in HT1080 cells. (A) HT1080 cells were subjected to increasing doses of type 1 - 4 FINs (erastin, RSL3, FIN56 and FINO2) and ferroptocide. The CellTiterGlo assay was used to detect adenosine triphosphate (ATP) luminescence. Dose-response regression curves are plotted. (B) HT1080 cells were treated with indicated doses of FIN56 for 24 h and 48 h. A representative set of primary data is depicted as well as a bar graph showing the mean +/- SD of double-negative events as assessed via flow cytometry analysis of 7-aminoactinomycin (7-AAD) and annexin V. (C) HT1080 cells were treated with FINs and ferroptocide upon increasing concentrations of Fer-1, Nec-1s and Nec-1f. ATP luminescence was measured and dose-response regression curves were plotted.

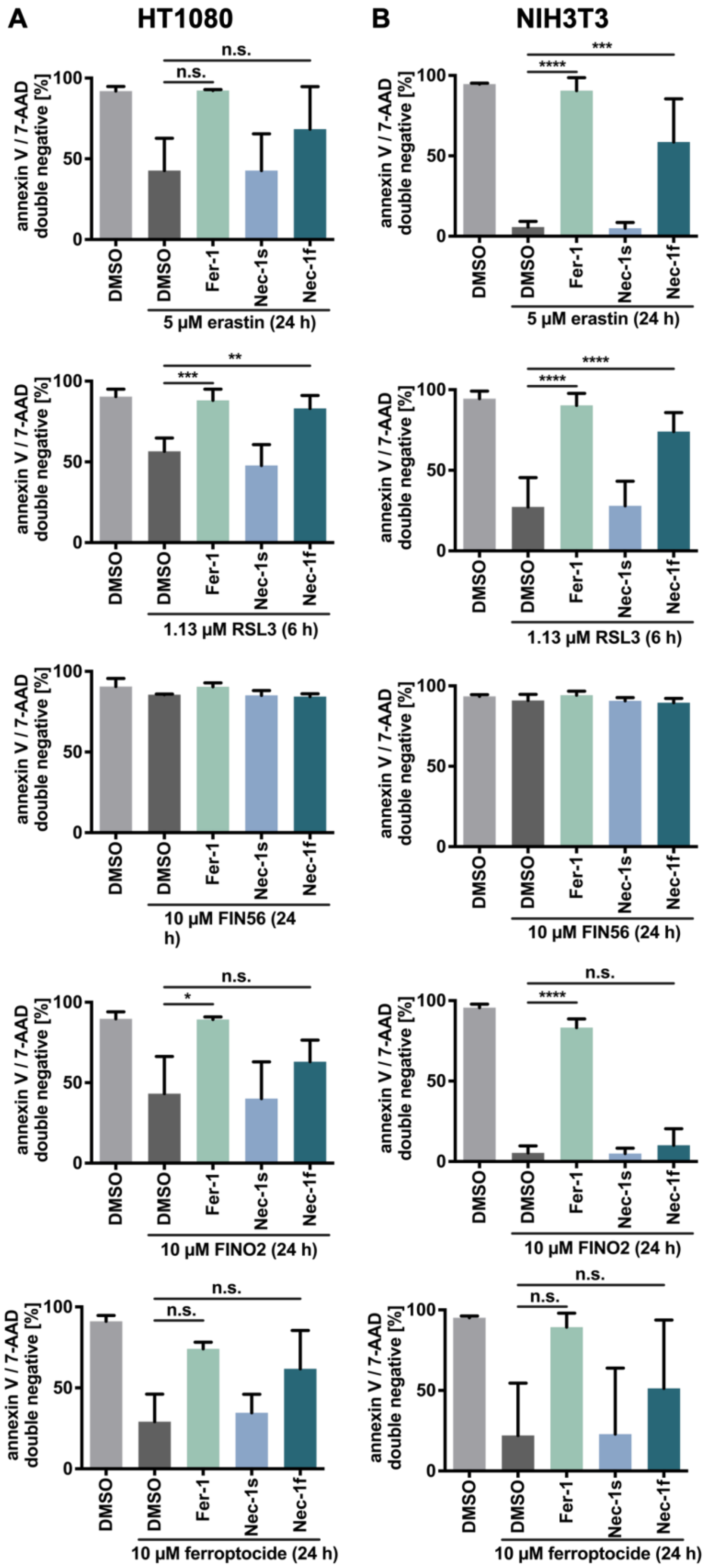

Figure S8: (corresponds to Figure 3): Nec-1f inhibits ferroptosis in HT1080 and NIH3T3 cells. (A) HT1080 cells were treated for 6 or 24 hours with FINs or ferroptocide in the presence of Fer-1, Nec-1s or Nec-1f, respectively. The bar graphs show the mean +/- SD of double-negative events. (B) NIH3T3 cells were treated and analyzed as in (A). Statistical analysis of at least three independent experiments was performed using one-way ANOVA. 7-AAD – 7-aminoactinomycin D

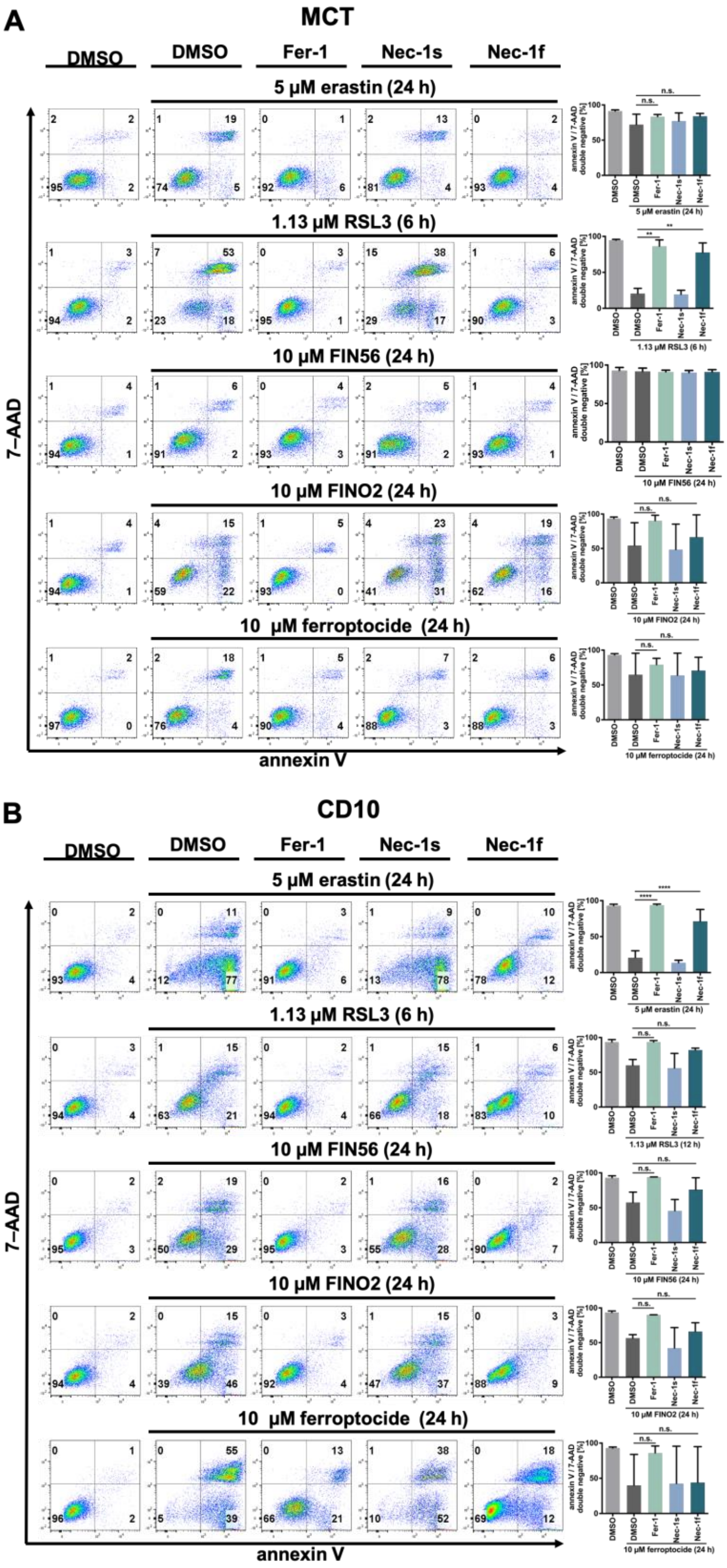

Figure S9: (A) Nec-1f prevents ferroptotic cell death in murine and human tubular cells. Mouse cortical tubular cells (MCTs) were treated with type 1-4 ferroptosis inducers (5 µM erastin, 1.13 µM RSL3, 10 µM FIN56, 10 µM FINO2) or 10 µM ferroptocide in the presence of Fer-1 (1 µM), Nec-1s (30 µM) or Nec-1f (30 µM) for indicated times (6 h in the case of RSL3, 24 h in all other cases). Cells were collected and stained for 7-aminoactinomycin (7-AAD) and annexin V. A representative set of primary data is demonstrated. The bar graphs show the mean +/- SD of double-negative events. (B) CD10-135 cells were treated and analyzed as in (A). Statistical analysis of three independent experiments was performed using one-way ANOVA.

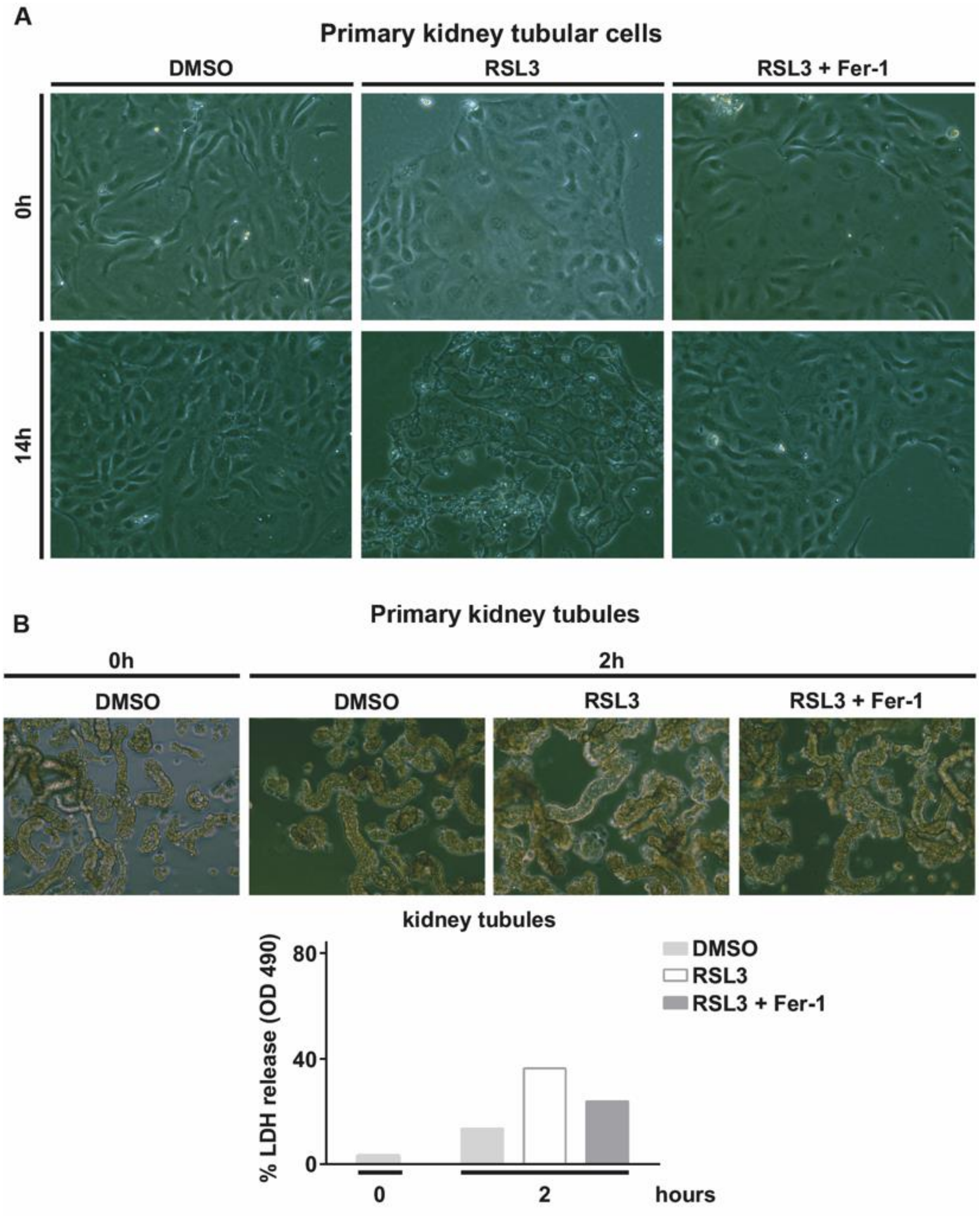

Figure S10: (associated with Figure 4). (A) Isolated renal tubules were maintained to allow outgrowth of primary tubular cells for 10 days. Representative high-resolution images are provided following treatment with a type 2 ferroptosis inducer (RSL3, 1.13 µM) in the presence of Fer-1 (1 µM), as indicated. Note the characteristic morphological change in the primary tubular cells treated with RSL3 for 14 hours, and the complete reversal of this phenotype by Fer-1. (B) Freshly isolated primary murine kidney tubules were treated for 2 hours with RSL3 (1.13 µM) with or without Fer-1 (1 µM). Representative images are presented and lactate dehydrogenase (LDH) release was quantified.

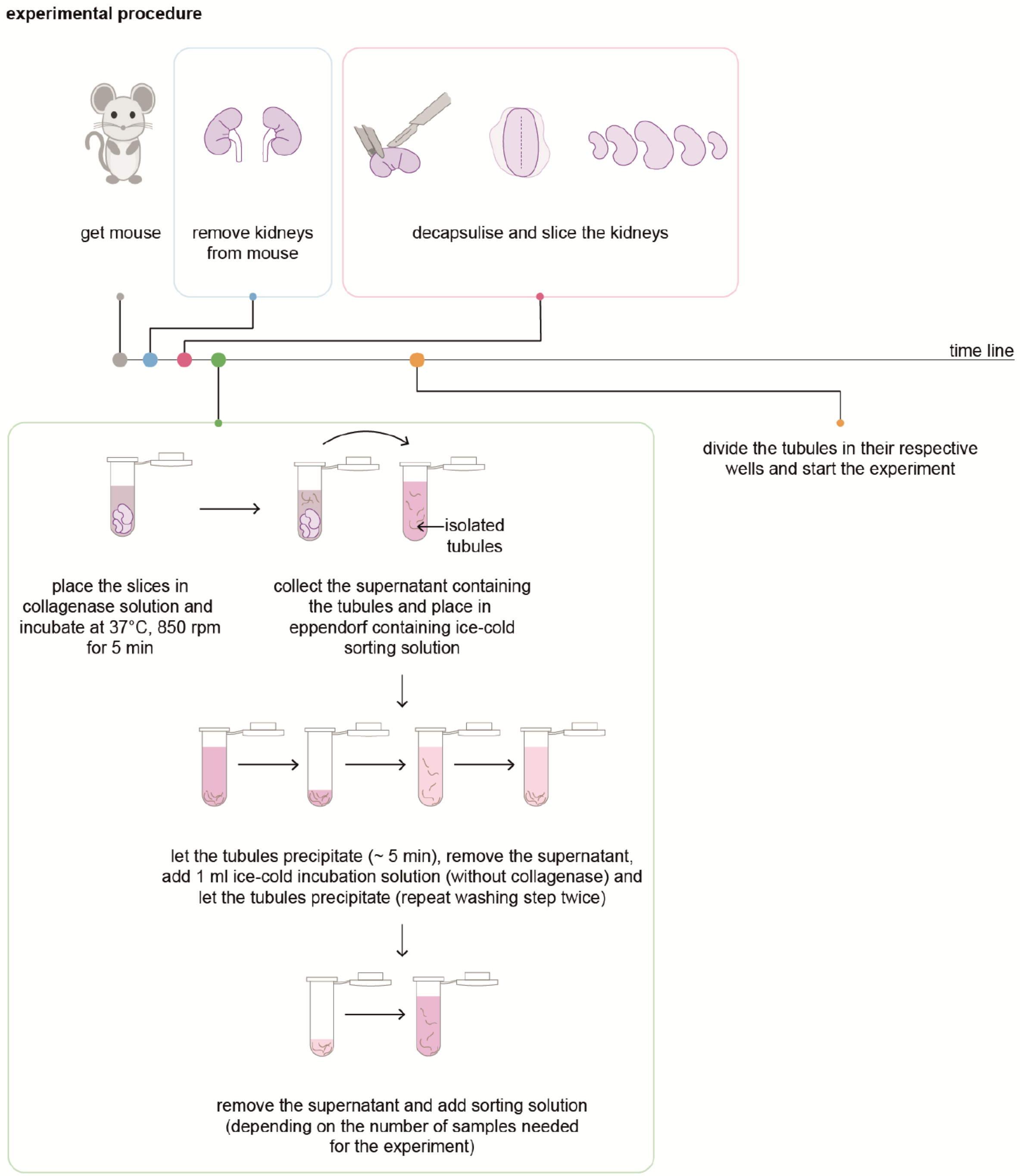

Figure S11: Protocol used for renal tubule isolation. The protocol for fresh primary kidney tubule isolation has been graphically explained. Please refer to the methods section for details. This scheme was illustrated by A.B.

A

substance	hepatic microsome stability: k_{el} [min^{-1}]	$t_{1/2}$ [min]	Cl_{int} [µl/ min/mg]	human plasma stability: $t_{1/2}$ [min]
diclofenac	0.105	6.6	253	
propanolol	0.021	33.0	51	
Nec-1f	0.027	25.7	65	**>120** (414)
propantheline				21
verapamil				**>120** (821)

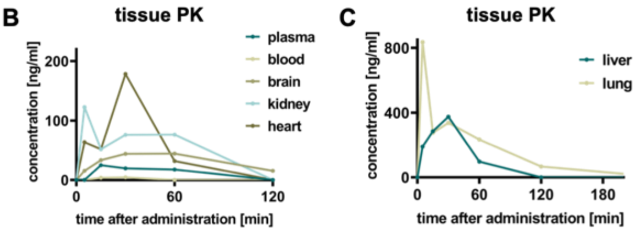

D

pharmacokinetic parameters

	t_{max} [min]	C_{max} [ng/ml]	$AUC_{0\rightarrow t\,min}$ [ng*min/ml]	$AUC_{0\rightarrow\infty}$ [ng*min/ml]	$t_{1/2}$ [min]	K_{el} [min^{-1}]
plasma	15	25.2	1.020	3.510	96.9	0.007
blood	BQL	BQL	BQL	ND	ND	ND
brain	60	44.4	3.980	4.830	38.9	0.018
liver	30	374	14.900	17.000	15.4	0.045
kidney	5	122	4.420	27.800	211.0	0.003
heart	30	178	5.450	6.010	12.1	0.058
lung	5	832	29.800	33.400	37.7	0.018
pancreas	60	55.700	4.050.000	4.080.000	66.3	0.011

Figure S12: Pharmakokinetic (PK) studies of Nec-1f i*n vitro* and mice. (A) Human hepatic microsomal stability for Nec-1f and human plasma stability of Nec-1f were assessed in comparison with reference substances (diclofenac, propanolol, propantheline, verapamil) over 120 min (corresponding to Figure 5). The elimination constant (k_{el}), half-life ($t_{1/2}$) and intrinsic clearance (Cl_{int}) in liver microsomes as well as the half life in human plasma values were determined in plot of ln(AUC) versus time, using linear regression analysis for all substances. (B - C) Levels of Nec-1f were determined by LC-MS/MS in the blood plasma, whole blood, brain, liver, kidney, heart, lung and pancreas over time after a single i.p. injection (2 mg/kg Nec-1f). Vehicle (n = 3) and Nec-1f (n = 27). (D) Assessment of pharmacokinetic parameters like the respective tissue half-life ($t_{1/2}$), the elimination constant (K_{el}) as well as time till maximum concentration (t_{max}) and the measured maximum concentration (c_{max}) of Nec-1f for each tissue. AUC = area under the curve.

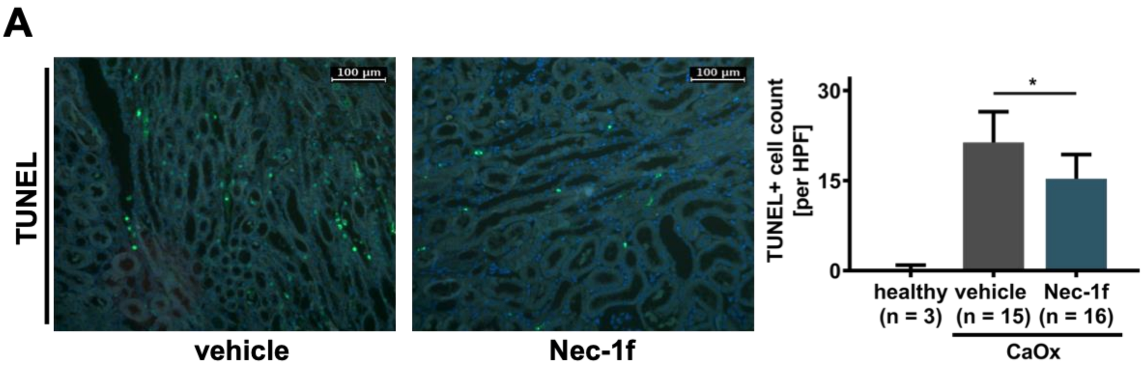

Figure S13 (associated with Figure 5): Nec-1f protects mice from CaOx-induced cell death. Mice received a single injection of 100 mg/kg sodium oxalate and were supplemented with 3 % sodium oxalate in the drinking water. TUNEL (TdT-mediated dUTP-biotin nick end labeling) staining visualizes DNA strand breaks in the kidneys of the mice 24 h after onset of calcium oxalate (CaOx) treament. The bar graph shows the mean +/- SD. Statistical analysis was performed using student´s t-test.

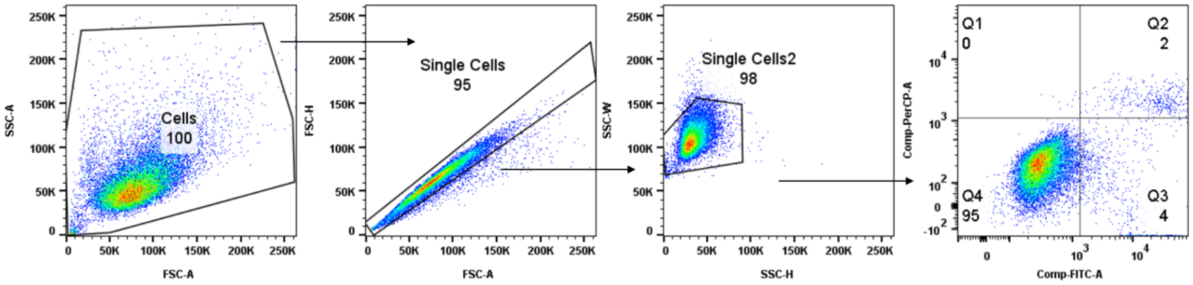

Figure S14: Gating strategy for flow cytometry data collection. Events were recorded and analyzed using the depicted gating scheme. Forward scatter (FSC-A) and side scatter (SSC-A) were used for selecting the cells, two distinct gates were set for exclusion of doublets and finally, FITC-A for detection of annexinV-FITC and PerCP-A for detection of 7-AAD were plotted. All plots shown in the manuscript figures were created using the depicted gating strategy. Only the final comparisons of FITC-A versus PerCP-A are shown in the manuscript figures.

Legends to Supplementary Movies

Supplementary Movie 1: Synchronized regulated necrosis of primary murine renal tubular cells. Time lapse of primary renal tubular cells undergoing RSL3-induced ferroptosis. Sytox green is used to visualize membrane permeability which we interpret as necrosis.

Supplementary Movie 2: Synchronized regulated necrosis of primary murine renal tubules. Time lapse of hand-picked, freshly isolated renal tubules undergoing spontaneous synchronized regulated necrosis.

Supplementary Movie 3: Neutrophil infiltration following cardiac allograft transplantation – control. Two photon intravital microscopy following cardiac allograft transplantation. Note that the recipient mouse carries LysM-GFP. Still images of this video are demonstrated in Figure 5G.

Supplementary Movie 4: Neutrophil infiltration following cardiac allograft transplantation – Nec-1f. Two photon intravital microscopy following cardiac allograft transplantation. Note that the recipient mouse carries LysM-GFP. Still images of this video are demonstrated in Figure 5G. In comparison with vehicle-treated mice, Nec-1f treatment resulted in lower numbers of extravasating cells.
