# Supplementary material for: Dysfunction of the key ferroptosis-surveilling systems hypersensitizes mice to tubular necrosis during acute kidney injury
Source: Nat Commun. 2021 Jul 20;12:4402. doi: 10.1038/s41467-021-24712-6 (PMC8292346; doi:10.1038/s41467-021-24712-6)
Supplement: Supplementary file 3 — Description of Additional Supplementary Files [file 41467_2021_24712_MOESM3_ESM.pdf]

**Description of Additional Supplementary Files**

File Name: Supplementary Movie 1
Description: Synchronized regulated necrosis of primary murine renal tubular cells. Time lapse of primary renal tubular cells undergoing RSL3-induced ferroptosis. Sytox green is used to visualize membrane permeability which we interpret as necrosis.

File Name: Supplementary Movie 2
Description: Synchronized regulated necrosis of primary murine renal tubules. Time lapse of hand-picked, freshly isolated renal tubules undergoing spontaneous synchronized regulated necrosis.

File Name: Supplementary Movie 3
Description: Neutrophil infiltration following cardiac allograft transplantation – control. Two photon intravital microscopy following cardiac allograft transplantation. Note that the recipient mouse carries LysM-GFP. Still images of this video are demonstrated in Figure 5G.

File Name: Supplementary Movie 4
Description: Neutrophil infiltration following cardiac allograft transplantation – Nec-1f. Two photon intravital microscopy following cardiac allograft transplantation. Note that the recipient mouse carries LysM-GFP. Still images of this video are demonstrated in Figure 5G. In comparison with vehicle-treated mice, Nec-1f treatment resulted in lower numbers of extravasating cells.
