# Supplementary material for: Dysfunction of the key ferroptosis-surveilling systems hypersensitizes mice to tubular necrosis during acute kidney injury
Source: Nat Commun. 2021 Jul 20;12:4402. doi: 10.1038/s41467-021-24712-6 (PMC8292346; doi:10.1038/s41467-021-24712-6)
Supplement: Supplementary file 8 — Reporting Summary [file 41467_2021_24712_MOESM8_ESM.pdf]

nature research

Corresponding author(s): Andreas Linkermann

Last updated by author(s): Jun 6, 2021

Reporting Summary

Nature Research wishes to improve the reproducibility of the work that we publish. This form provides structure for consistency and transparency in reporting. For further information on Nature Research policies, see our Editorial Policies and the Editorial Policy Checklist.

Statistics

For all statistical analyses, confirm that the following items are present in the figure legend, table legend, main text, or Methods section.

n/a	Confirmed	
☐	☒	The exact sample size (*n*) for each experimental group/condition, given as a discrete number and unit of measurement
☐	☒	A statement on whether measurements were taken from distinct samples or whether the same sample was measured repeatedly
☐	☒	The statistical test(s) used AND whether they are one- or two-sided<br>*Only common tests should be described solely by name; describe more complex techniques in the Methods section.*
☒	☐	A description of all covariates tested
☒	☐	A description of any assumptions or corrections, such as tests of normality and adjustment for multiple comparisons
☐	☒	A full description of the statistical parameters including central tendency (e.g. means) or other basic estimates (e.g. regression coefficient) AND variation (e.g. standard deviation) or associated estimates of uncertainty (e.g. confidence intervals)
☐	☒	For null hypothesis testing, the test statistic (e.g. *F*, *t*, *r*) with confidence intervals, effect sizes, degrees of freedom and *P* value noted<br>*Give P values as exact values whenever suitable.*
☒	☐	For Bayesian analysis, information on the choice of priors and Markov chain Monte Carlo settings
☒	☐	For hierarchical and complex designs, identification of the appropriate level for tests and full reporting of outcomes
☒	☐	Estimates of effect sizes (e.g. Cohen's *d*, Pearson's *r*), indicating how they were calculated

Our web collection on statistics for biologists contains articles on many of the points above.

Software and code

Policy information about availability of computer code

Data collection	Flow cytometry: For the sample collection the BDTM LSRII and the FACS Diva 6.1.1 software (BD Biosciences) was used. For data analysis the FlowJo v10 software was used (Tree Star).<br>LDH release: The LDH release (Absorbance at 490 nm) was recorded using a TECAN multiplate reader.<br>CellTiter-Glo® Assay: Luminescence was recorded using a Promega GloMax® instrument.<br>Histology: ZEN 2.1 SP1 (Zeiss) software was used for taking images.<br>Live cell imaging: Images were recorded using an Axiovert 200M (Zeiss) and the VisiView® software v 4.4.0.0. (Visitron Systems).<br>Inhibited co-autoxidation: A BioTek SynergyTM H1 plate reader was used to record the emission was measured at 518 nm.<br>Assessment of metabolic stability: Supernatants were analyzed using the HPLC system coupled with tandem mass spectrometer API 3000TM (AB Sciex).<br>Western blotting: detection by AMERSHAMTM ImageQuantTM 800.<br>Cell death in primary murine tubular cells: Images of the treated primary murine tubular cells were obtained using a 20x/0.30 PH1 objective on a Leica DMi1 (Leica Microsystems) microscope.<br>Intravital imaging: Intravital two-photon imaging was performed using a custom-built microscope running ImageWarp version 2.1 acquisition software (A&B Software).<br>Tissue PK studies: Measurements were performed using the Shimadzu Prominence HPLC system including vacuum degasser, gradient pumps, reverse phase HPLC column, column oven, and autosampler. Mass spectrometric analysis was performed using an API 4.000 QTRAP mass spectrometer from Applied Biosystems/ MDS Sciex (AB Sciex) with Turbo V ion source and TurboIonSpray® interface. The data acquisition and system control was performed using Analyst 1.6.3 software from AB Sciex.
Data analysis	Data analysis and graph plotting: Microsoft Excel 365 MSO (16.0.13127.20266) 32-Bit, GraphPad Prism 8 (v8) were used for data analysis.<br>Flow cytometry: FlowJo v10 software (Tree Star) was used for the analysis of the recorded events at the flow cytometer.<br>Live cell imaging: FIJI (Version 1.51c) was used for creating the videos.

Tissue PK studies: The pharmacokinetic data analysis was performed using WinNonlin 5.2 (PharSight). Data analysis from mass spectrometry was performed using Analyst® 1.6.3 software from AB Sciex.
Intravital imaging: Imaris (Bitplane) was used for multidimensional rendering and manual cell tracking.

For manuscripts utilizing custom algorithms or software that are central to the research but not yet described in published literature, software must be made available to editors and reviewers. We strongly encourage code deposition in a community repository (e.g. GitHub). See the Nature Research guidelines for submitting code & software for further information.

Data

Policy information about availability of data

All manuscripts must include a data availability statement. This statement should provide the following information, where applicable:
- Accession codes, unique identifiers, or web links for publicly available datasets
- A list of figures that have associated raw data
- A description of any restrictions on data availability

The data that support the findings of this study are available from the corresponding author upon reasonable request.

Field-specific reporting

Please select the one below that is the best fit for your research. If you are not sure, read the appropriate sections before making your selection.

☒ Life sciences ☐ Behavioural & social sciences ☐ Ecological, evolutionary & environmental sciences

For a reference copy of the document with all sections, see nature.com/documents/nr-reporting-summary-flat.pdf

Life sciences study design

All studies must disclose on these points even when the disclosure is negative.

Sample size	For all animal experiments, a calculation of group sizes was performed as required by the German law. For values of serum creatinine/urea, an estimated mean and standard deviation of both groups was used based on previous experiments. We used a margin of alpha error of 5% and beta error of 80 % to detect an estimated difference of 25%. For survival analysis, estimated survival rates and a 7-day observation period were used to calculate group sizes. Again, a margin of alpha 5% and beta error of 80% was used. As log-rank was the desired statistics, repetitive simulations were performed to determine group sizes. For severe IRI with Nec-1f, we calculated for a survival of 50 % in the vehicle group after 5 days. All these statistical presumptions were chosen on 10 years of experience with these models and a corresponding load of predata.
Data exclusions	In ischemia/reperfusion experiments, data of single mice were excluded in the rare event of severe surgical complications (e.g. bleeding, insufficient vessel clamping or delayed reperfusion). Severe surgical complications did not occur in any of the specimens presented here.
Replication	All animal experiments were performed according to the n of each experiment. A (n = 8) means that the identical surgical procedure was performed in 8 mice in this group. Mouse experiments presented in this study were not repeated as each single mouse represents a repetition of a complex experiment; thus, in a n = 8 experiments, the data of 8 repetitions are presented. For cell culture experiments, at least n = 2 replicates per treatment were performed and, depending on the variability of the positive and negative controls, up to n = 8 analyses were performed.
Randomization	n/a - genetically modified mice were matched to wildtypes.
Blinding	All experiments featuring mice were conducted in a strictly blinded manner. The surgeon was unaware of the genotype and/or drug the mouse recieved. Furthermore, all histopathological evaluations were performed in a strictly blinded manner.

Reporting for specific materials, systems and methods

We require information from authors about some types of materials, experimental systems and methods used in many studies. Here, indicate whether each material, system or method listed is relevant to your study. If you are not sure if a list item applies to your research, read the appropriate section before selecting a response.

Materials & experimental systems

n/a	Involved in the study	
☐	☒	Antibodies
☐	☒	Eukaryotic cell lines
☒	☐	Palaeontology and archaeology
☐	☒	Animals and other organisms
☒	☐	Human research participants
☒	☐	Clinical data
☒	☐	Dual use research of concern

Methods

n/a	Involved in the study	
☒	☐	ChIP-seq
☐	☒	Flow cytometry
☒	☐	MRI-based neuroimaging

Antibodies

Antibodies used

Rabbit monoclonal anti-RIPK1 (phospho S166), human: Cell Signaling Cat# 65746
Rabbit monoclonal anti-MLKL (phospho S358), human: Abcam Cat# ab187091
Rabbit polyclonal anti-MLKL, human: Genetex Cat# GTX107538
β-actin: Cell Signaling Cat# 3700S
Anti-mouse IgG, HRP-linked antibody: Cell Signaling Cat# 7076S
Anti-rabbit IgG, HRP-linked antibody: Cell Signaling Cat# 7074S
Anti-4 Hydroxynonenal antibody: Abcam Cat# ab46545

Validation

Antibodies were tested in a variety of cell lines and/or crCas9-knockout cell lines, and respective positive controls were used in all experiments.
Anti-4 Hydroxynonenal antibody: Abcam Cat# ab46545
Specificity: Specifically binds to HNE modified proteins.
Tested applications: WB, ELISA
Species reactivity: Reacts with: Species independent
Selected publications:
PubMed: 32145212
PubMed: 33279868
PubMed: 32027621
PubMed: 32769011

Rabbit monoclonal anti-RIPK1 (phospho S166), human: Cell Signaling Cat# 65746
Specificity / Sensitivity: Phospho-RIP (Ser166) (D1L3S) Rabbit mAb recognizes endogenous levels of RIP protein only when phosphorylated at Ser166.
Species Reactivity: Human
Source / Purification: Monoclonal antibody is produced by immunizing animals with a synthetic phosphopeptide corresponding to residues surrounding Ser166 of human RIP protein.
Selected publications:
Meylan, E. and Tschopp, J. (2005) Trends Biochem Sci 30, 151-9.
Kaczmarek, A. et al. (2013) Immunity 38, 209-23.
Degterev, A. et al. (2008) Nat Chem Biol 4, 313-21.
Degterev, A. et al. (2005) Nat Chem Biol 1, 112-9.
Ofengeim, D. and Yuan, J. (2013) Nat Rev Mol Cell Biol 14, 727-36.

Rabbit monoclonal anti-MLKL (phospho S358), human: Abcam Cat# ab187091
Specificity / Sensitivity: Rabbit monoclonal [EPR9514] to MLKL (phospho S358). Stimulation may be required to allow detection of the phosphorylated protein.
Tested applications: WB, Dot blot, IHC-P
Species Reactivity: Human
Selected publications:
PubMed: 31894265
PubMed: 31914150
PubMed: 31969562
PubMed: 32051018
PubMed: 32075957

Rabbit polyclonal anti-MLKL, human: Genetex Cat# GTX107538
Specificity / Sensitivity: Rabbit polyclonal MLKL
Tested applications: WB, IP
Species Reactivity: Human, Mouse
Selected publications:
Johnston AN et al. Proc Natl Acad Sci U S A 2020; 117 (12):6521-6530 Necroptosis-blocking compound NBC1 targets heat shock protein 70 to inhibit MLKL polymerization and necroptosis.
Hanna-Addams S et al. Proc Natl Acad Sci U S A 2020; 117 (4):1962-1970 CK1α, CK1δ, and CK1ε are necrosome components which phosphorylate serine 227 of human RIPK3 to activate necroptosis.
Seo J et al. Cell Death Differ 2020; (Epub) Beclin 1 functions as a negative modulator of MLKL oligomerisation by integrating into the necrosome complex.
Dai J et al. Cell Death Dis 2020; 11 (4):282 A necroptotic-independent function of MLKL in regulating endothelial cell adhesion molecule expression.

β-actin: Cell Signaling Cat# 3700S
Specificity / Sensitivity: β-Actin (8H10D10) Mouse mAb detects endogenous levels of total β-actin protein. Due to the high sequence identity between the cytoplasmic actin isoforms, β-actin and cytoplasmic γ-actin, this antibody may cross-react with cytoplasmic γ-actin. It does not cross-react with α-skeletal, α-cardiac, α-vascular smooth, or γ-enteric smooth muscle isoforms.
Tested applications: WB, IHC, IF, F
Species Reactivity: Human, Mouse, Rat, Hamster, Monkey, Dog
Selected publications:

Herman, I.M. (1993) Curr. Opin. Cell Biol. 5, 48-55.
Perrin, B.J. and Ervasti, J.M. (2010) Cytoskeleton (Hoboken) 67, 630-4.
Condeelis, J. (2001) Trends Cell Biol 11, 288-93.
Lim, Y.P. et al. (2004) Clin Cancer Res 10, 3980-7.

Eukaryotic cell lines

Policy information about cell lines

Cell line source(s)	Murine NIH3T3 and human HT1080 cell lines were purchased from the American Type Culture Collection (ATCC, Manassas, VA, USA). Mouse cortical tubule cells (MCT) were kindly provided by Alberto Ortiz, which is the original source of this cell line. HT29 cells were kindly provided by Simone Fulda, who purchased this cell line from ATCC . Immortalized human kidney tubular epithelial CD10-135 cells were established in the Rafael Kramann laboratory (which is the original source of this cell line) and kindly provided for this project.
Authentication	Cell lines were not externally authenticated.
Mycoplasma contamination	All cell lines used were tested negative for mycoplasma contamination.
Commonly misidentified lines (See ICLAC register)	None of the cell lines used in this study are registered in the ICLAC register.

Animals and other organisms

Policy information about studies involving animals; ARRIVE guidelines recommended for reporting animal research

Laboratory animals	All mice used in this study were kept under stable 12-hour cirlces of darkness and light in the respective facilities. Room temperature was kept between 20 - 24 °C and air humidy between 45 - 65% as documented in daily controls. If not otherwise indicated, all cages were IVCs which fulfilled at least Euonorm type II. Mice had access to sterilized standart pellet food and water ad libidum. All cages and nestlets were sterilized by autoclaving before use.<br>All mice used for ischemia/reperfusion injury were males aged 8-12 weeks. For cisplatin-induced AKI, females aged 8-12 weeks were used. All controls were rigorously matched to their counterparts. As wildtypes we used C57Bl/6N mice purchased from Charles River Laboratories (Sulzfeld, Germany). GPX4cys/- mice and corresponding GPX4wt littermates were reported previously and were kindly provided by Marcus Conrad. FSP1-ko mice (AIFM2-ko; (B6.129-Aifm2tm1Marc/leg)) and corresponding wild type littermates were also kindly provided by Marcus Conrad. All provided mice were unblinded to their respective genotype only after all data were collected.<br>For acute oxalate nephropathy, C57BL/6N mice were procured from Charles River Laboratories (Sulzfeld, Germany) and co-housed in groups of four in filter top cages with unlimited access to food and water at the mouse facility of the Unviersity Hospital of the LMU Munich.<br>Sex-matched, 6-10 week-old C57BL/6 (The Jackson Laboratory, Bar Harbor, ME) and C57BL/6 LysM-GFP mice (provided by M. Miller, Washington University in St. Louis and originally obtained from K. Ley, La Jolla Institute for Allergy and Immunology, La Jolla, CA) were utilized for intravital imaging of transplanted hearts.
Wild animals	The study did not involve wild animals.
Field-collected samples	No samples were collected from the field.
Ethics oversight	All experiments featuring mice were performed according to German animal protection laws and were approved by ethics committees and local authorities in Kiel (Germany) and Dresden (Germany). For Kiel, this was the ethics comittee of the CAU Kiel and the Umwelt- und Landwirtschaftsministerium Schleswig-Holstein, respectively, whereas in Dresden, the ethics comitee of the TU Dresden and the Landesdirektion Sachsen approved the protocol.<br>Heart transplant experiments were approved by the Institutional Animal Studies Committee at Washington University.<br>Acute oxalate nephropathy models were approved by the Regierung von Oberbayern, München, Germany (ROB-55.2-2532.Vet_02-18-127).

Note that full information on the approval of the study protocol must also be provided in the manuscript.

Flow Cytometry

Plots

Confirm that:

- [x] The axis labels state the marker and fluorochrome used (e.g. CD4-FITC).
- [x] The axis scales are clearly visible. Include numbers along axes only for bottom left plot of group (a 'group' is an analysis of identical markers).
- [x] All plots are contour plots with outliers or pseudocolor plots.
- [x] A numerical value for number of cells or percentage (with statistics) is provided.

Methodology

Sample preparation	Cells were harvested and the pellets were washed twice in PBS and stained with 5 µl of 7-AAD (BD Biosciences) and 5 µl of annexin-V-FITC (BD Biosciences) added to 100 µl annexin-V binding buffer (BD Biosciences). After 15 min, cells were recorded on the LSRII with the FACS Diva 6.1.1 software (BD Biosciences) and subsequently analyzed with the FlowJo v10 software (Tree Star).
Instrument	LSRII (BD Biosciences)
Software	Events were recorded with the FACS Diva 6.1.1 software (BD Biosciences) and subsequently analyzed with the FlowJo v10 software (Tree Star).
Cell population abundance	Approximately 300.000 cells were collected per treatment and cells were gated to exclude doublets. In the controls, the double-negative population was usually higher than 90 %.
Gating strategy	FSC/SSC was used to set an original gate ifor our cell population. We did not exclude dead cells / debris from this gate. The following 2 sub-gates were added to exclude doublets and ultimately the single cell gate was then used to record 10.000 events per sample. Those were anaysed for 7-AAD and annexinV (FITC) positivity.

☒ Tick this box to confirm that a figure exemplifying the gating strategy is provided in the Supplementary Information.
